# Does Fish Oil Have an Anti-Obesity Effect in Overweight/Obese Adults? A Meta-Analysis of Randomized Controlled Trials

**DOI:** 10.1371/journal.pone.0142652

**Published:** 2015-11-16

**Authors:** Shichun Du, Jie Jin, Wenjun Fang, Qing Su

**Affiliations:** Department of Endocrinology, Shanghai Xin Hua Hospital affiliated to Shanghai Jiao Tong University School of Medicine, Shanghai, China; Medical University Innsbruck, AUSTRIA

## Abstract

**Context:**

Accumulating evidence has suggested favorable effects of fish oil on weight loss in animal experiments; however, findings remain inconsistent in humans.

**Objects:**

The meta-analysis was performed to investigate the influence of fish oil on some parameters of body composition in overweight/obese adults.

**Design:**

Human randomized, placebo-controlled trials were identified by a systematic search of Embase, PubMed, the Cochrane Library, web of science and reference lists of related reviews and articles. The random-effects model was used to estimate the calculated results.

**Results:**

In total, 21 studies with 30 study arms were included in this analysis. Calculated results of the meta-analysis demonstrated that fish oil had no effect on reducing body weight (overall SMD = -0.07, 95% CI -0.21 to 0.07, *P* = 0.31) and BMI (overall SMD = -0.09, 95% CI -0.22 to 0.03, *P* = 0.14) whether alone or combined with life modification intervention in overweight/obese subjects. However, waist circumference was significantly reduced (SMD = -0.23, 95% CI -0.40 to -0.06, *P* = 0.008) in those with fish oil supplementation combined with life modification intervention. Waist hip ratio (WHR) was significantly reduced (overall SMD = -0.52 95% CI -0.76 to -0.27, *P* < 0.0005) in fish oil supplemented individuals with or without combination life modification intervention.

**Conclusion:**

Current evidence cannot support an exact anti-obesity role of n-3 polyunsaturated fatty acids (PUFAs) in overweight/obese subjects. However, these subjects may benefit from reducing abdominal fat with fish oil supplementation especially when combined with life modification intervention. Further large-scale and long-term clinical trials are needed to gain definite conclusions.

## Introduction

The prevalence of overweight/obese humans worldwide brings an enormous risk of metabolic and cardiovascular diseases as well as large healthcare costs. Fish oil is associated with a body weight/fat reduction effect in high fat diet-fed obese animal models [1–4]. The potential anti-obesity mechanisms involved in fish oil have been proposed in these studies, including increased adipocyte apoptosis [2], increased plasma adiponectin levels [3, 5], and altered fat oxidation and energy expenditure [4, 6]. However, it remains unknown whether fish oil consumption can combat obesity in humans though there is emerging evidence that it can improve lipid profile [7–9] and cardiovascular function [10] in many human trials. The results of previous randomized controlled trials on possible weight reduction effects of fish oil in overweight/obese human are controversial, partly because of the limited number of included subjects or varied intervention methods. Therefore, this meta-analysis aimed to systematically examine the possible anti-obesity effects of fish oil with/without combination of life modification intervention in overweight/obese adults.

## Method and Search Strategy

This meta-analysis was performed in accordance with Preferred Reporting Items for Systematic Reviews and Meta-Analyses guidance [11]. We searched electronic databases in Embase, PubMed, web of knowledge, and the Cochrane Library for relevant records using the terms “fish-oil”, “fish oil”, “ducosahexaenoic acid”, “DHA”, “eicosapentaenoic acid”, “EPA”, and “n-3 polyunsaturated fatty acids”. These terms were paired with the vocabulary “body composition”, “obese”, “overweight”, “weight loss”, “weight reduction”, or “obesity”. The search was limited to human randomized and placebo-controlled studies. Meanwhile, we manually searched reference lists of relevant review or original articles.

### Study selection criteria

Studies were included for analysis if they qualified the following criteria: 1) they were reported as a prospective, randomized (parallel or crossover), blind (double or single) and controlled trials, regardless of sample size; 2) they analyzed human subjects (≥18 y of age and body mass index (BMI) ≥25) who were assigned to either fish oil /marine diet or a control group for ≥4 weeks; 3) reported at least one of the following body composition parameters including body weight, BMI, waist circumference, and waist/hip ratio (WHR).

### Data extraction and quality assessment

The literature search and data extraction were performed independently by two authors (S.D. and J.J.) according to standardized inclusion criteria with standardized individual results. Discrepancies were resolved by discussion with a third author (W.F.).The extracted data included the following parts: study design characteristics (crossover or parallel, double or single blind), participants characteristics (number, sex, age, and general health status), intervention strategies (dose of n-3 PUFA, ratio of EPA to DHA, treatment options in control groups, and combination with life modification intervention), study duration, reported changes in the body composition measurement (changes of body weight, BMI, waist circumstance, and WHR). For trials with more than one intervention group (with different doses of fish oil or with different intervention), multiple comparisons were considered. An assessment of study quality was performed independently by S.D. and J.J. according to the quality scores proposed by Jadad et al [12]. Some authors were contacted by email for original data if they were not available in the literatures.

### Statistical analysis

Analysis endpoints were calculated based upon the changes from baseline to post-treatment. Pooled effects were reported as standardized mean differences (SMDs) and 95% confidence intervals (CIs). The Cochrane's test was used to assess inter-study heterogeneity. Heterogeneity was identified as significant among the trials if the *P* value was <0.10, or I^2^ > 50% [13]. A random-effects model was used to estimate the pooled effects, which provided more generalized results. Furthermore, the predefined subgroup analyses were performed to explore the possible influence of study characteristics on study outcomes. Sensitivity analyses were also performed by omitting the studies with short follow-up duration. Egger regression asymmetry tests [14] and funnel plots were used to assess potential publication bias. *P* values were 2-tailed with statistical significance set at 0.05. Statistical analyses were performed with Stata software (version 12.0; Stata Corp).

## Results

### Search results

**Fig 1:**
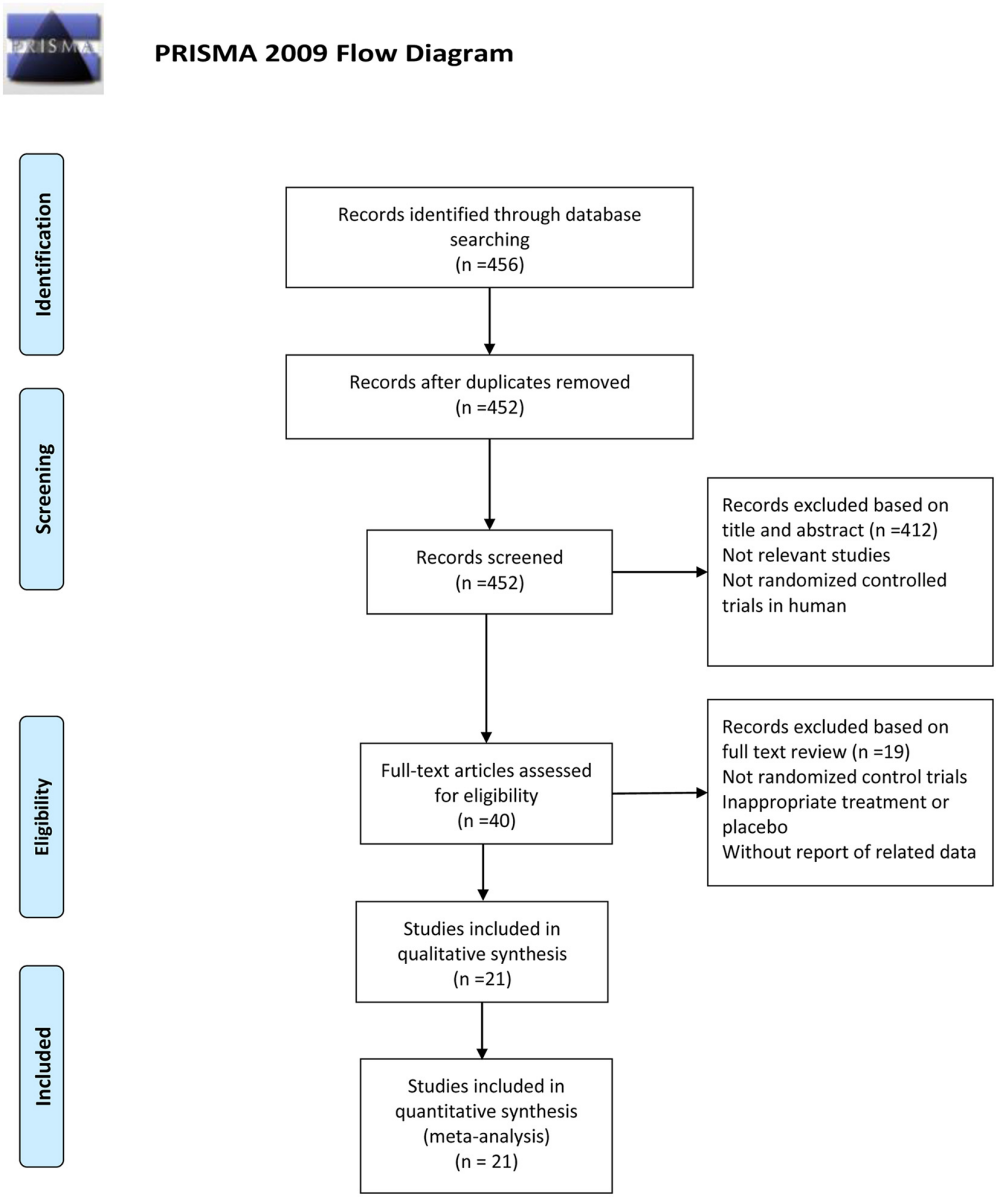
PRISMA 2009 flow diagram illustrates the study selection procedure. *From*: Moher D, Liberati A, Tetzlaff J, Altman DG, The PRISMA Group (2009). *P*referred *R*eporting *I*tems for *S*ystematic Reviews and *M*eta-*A*nalyses: The PRISMA Statement. PLoS Med 6(6): e1000097. doi:10.1371/journal.pmed1000097. **For more information, visit** www.prisma-statement.org.

In total, 456 articles were initially identified through the database search. Of these, 4 records were duplicated and 412 records were excluded because the objectives of these studies were irrelevant to the current meta-analysis, or because lacking randomization and control. Of the 40 potentially relevant records identified, 21 [9, 15–34] met the inclusion criteria for this meta-analysis. 19 were excluded because 3 were not randomized controlled trials [8, 35, 36], 1 compared fish diet with fish oil supplements [37], 1 compared the mixture of linoleic acid and n-3 PUFAs with placebo [38], 1 did not provide n-3 PUFAs dosage [39], 2 had a treatment duration of only 3 weeks [40, 41], 3 included infants or children [42–44], 7 did not provide related body compositions data [35, 45–50], and 1 did not report the number of both groups [51] (Fig 1).

### Study characteristics

Data extracted from 1652 individuals who participated in the 21 studies were analyzed in this meta-analysis (Table 1). The studies investigated by Hill et al [18], Mori et al [26], and Munro et al [27] included two randomized comparisons in participants who underwent both fish oil alone and combined with a weight loss program. The studies investigated by Thorsdottir et al [30], Sjoberg et al [29], and Crochemore et al [21] included two randomized comparisons in participants who were treated with two different doses of n-3 PUFAs. Finally, 30 sets of data comparing fish oil treatment with controls were enrolled. All of the included comparisons were randomized controlled trials with 23 in a double-blind and 7 in a single-blind design. All of the studies included subjects of 18 years or older who were overweight or obese. Fish oil capsules were supplied orally in all of the studies except one, which fed marine food to the treatment group [30]. For the convenience of data pooling, the "high dose fish oil" group was selected as the "treatment" group while the "low dose fish oil" was selected as the control group for analysis in the study of Moore et al [9]. Overall, the median n3-PUFAs dose was 1.92 g/day (range: 0.54–11.3 g/day), and the ratio of EPA to DHA varied from 0.23 to 1.55. One study used highly purified EPA only [20]. Median treatment duration with n-3 PUFAs was 12 weeks (range: 4–24 weeks). For those with life modification intervention, subjects were given dietary caloric restriction in eleven studies, both caloric restriction and physical exercise in one study [16] or physical exercise only in one study [18].

**Table 1:** Overview and characteristics of included studies.

Study	Design	Subjects number	Age y	Male %	BMI	General health	Total dose g/d	EPA/DHA	Duration wk	Control number	Control oil	Dropout %	Body composition parameters reported	Life style modification	Quality score
Mori et al, 1999a	Parallel	33	54	73	33	hypertension	3.65	NR	16	16	NR	NR	Wt BMI WC WHR	no	1
Moore et al, 2006a	single blind	55	51	35	31	Healthy	0.64	NR	24	24	low fish oil	6	Wt WC	no	3
Moore et al, 2006b	single blind	57	51	35	31	Healthy	0.64	NR	24	30	low fish oil	6	Wt WC	no	3
Hill et al,2007a	Parallel	38	51	32	34	cardiovascular risk factor	1.92	0.23	6	20	sunflower oil	13	Wt BMI	no	5
Kabir et al, 2007	Parallel	26	55	0	30	T2DM	1.8	1.5	8	14	paraffin oil	NR	Wt	no	5
Itoh et al, 2007	single blind	52	52	41	30	Healthy	1.8	only EPA	12	26	saline	NR	BMI WC	no	1
Cussons et al,2009	Crossover	25	33	0	35	PCOS	3.32	0.48	8	25	olive oil	0	BMI WHR	no	2
Hartwich et al,2009	Parallel	41	55	34	35	metabolic syndrome	1.24	NR	12	22	sunflower oil	NR	BMI	no	2
Sjoberg et al,2010a	Parallel	33	53	54	32	Healthy	0.64	0.23	12	17	sunola oil	17	BMI	no	3
Sjoberg et al,2010b	Parallel	34	53	54	32	Healthy	1.28	0.23	12	17	sunola oil	17	BMI	no	3
Sjoberg et al,2010c	Parallel	34	53	54	32	Healthy	1.92	0.23	12	17	sunola oil	17	BMI	no	3
Tierney et al,2011	single blind	206	55	44	32	Met S	1.24	NR	12	106	sunflower oil	14	Wt BMI WC	no	3
Vargas et al,2011	Parallel	34	30	0	34	PCOS	3.5	1.48	6	17	soybean oil	18	Wt BMI WC	no	3
Gammelmark et al, 2012	Parallel	50	57	48	30	Healthy	1.12	1.33	6	24	olive oil	2	BMI	no	3
Itariu et al,2012	Parallel	55	38	16	46	Severely obese	3.36	1.21	8	28	butterfat	11	Wt BMI WHR	no	3
Rafraf et al, 2012	Parallel	64	27	0	29	PCOS	1.2	1.5	8	31	paraffin oil	5	Wt BMI WHR WC	no	5
Crochemore et al,2012a	single blind	27	61	0	31	T2DM	0.54	1.55	4	13	gelatin	NR	BMI WC	no	2
Crochemore et al,2012b	single blind	27	61	0	31	T2DM	0.9	1.55	4	13	gelatin	NR	BMI WC	no	2
Munro et al, 2013a	Parallel	39	46	23	32	Healthy	2.04	0.26	4	19	sunola oil	7	Wt BMI WHR WC	no	5
Mori et al, 1999b	Parallel	30	54	60	33	hypertension	3.65	NR	16	16	NR	NR	Wt BMI WC WHR	diet	1
Mendez-Sanchez et al, 2001	Parallel	35	38	0	34	Healthy	11.3	NR	6	11	NR	0	Wt BMI	diet	3
Krebs et al,2006	Parallel	93	45	0	35	hyperinsulinemia	4.2	0.45	24	26	linoleic and oleic acid	20	Wt BMI WC	diet	3
Hill et al,2007b	Parallel	37	51	32	34	cardiovascular risk factor	1.92	0.23	12	18	sunflower oil	13	Wt BMI BF	exercise	5
Thorsdottir et al, 2007a	Parallel	140	31	43	30	Healthy	3	NR	8	66	sunflower oil	7	Wt BMI WHR WC	diet	3
Thorsdottir et al, 2007b	Parallel	134	31	43	30	Healthy	1.5	NR	8	66	sunflower oil	14	Wt BMI WHR WC	diet	3
Kratz et al,2008	Parallel	26	38	39	30	Healthy	2.88	NR	14	13	NR	21	Wt BMI	diet	2
DeFina et al,2011	Parallel	128	46	31	33	Healthy	3	5	24	64	soybean/corn oil	4	Wt BMI WC	diet and exercise	5
Munro et al, 2013b	Parallel	39	46	23	32	Healthy	2.04	0.26	8	19	sunola oil	7	Wt BMI WHR WC	diet	5
Munro et al,2013c	Parallel	35	40	NR	33	Healthy	2.04	0.26	12	18	sunola oil	23	Wt BMI WHR WC	diet	5
Wong et al, 2013	single blind	25	60	56	33	Healthy	3.36	1.21	16	12	none	7	Wt BMI WHR WC	diet	2

NR, not reported; PCOS, polycystic ovary syndrome; T2DM, type 2 diabetes; Met S, metabolic syndrome; Wt, body weight; BMI, body mass index; WHR, waist hip ratio; WC, waist circumference.

General health, overall health status apart from overweight/obese.

The study by Thorsdottir et al had a fish oil capsules group (1.5 g/d EPA + DHA) and fatty fish diet group (3g/d EPA+DHA).

The study by Crochemore et al was given fish oil capsules with 0.54 or 0.9 g/d EPA +DHA.

The study by Sjoberg et al was given fish oil capsules with 0.64, 1.28, or 1.92g/d EPA +DHA.

The study by Moore et al compared 4.5g/week EPA+DHA plus rapeseed oil with 0.7g/week EPA+DHA plus rapeseed oil; 4.5g/week EPA+DHA plus sunflower oil with 0.7g/week EPA+DHA plus sunflower oil.

The studies by Mori, Hill, and Munro et al included comparisons with participants underwent fish oil alone or combined with weight loss program.

### Effects of fish oil on body weight changes

**Fig 2:**
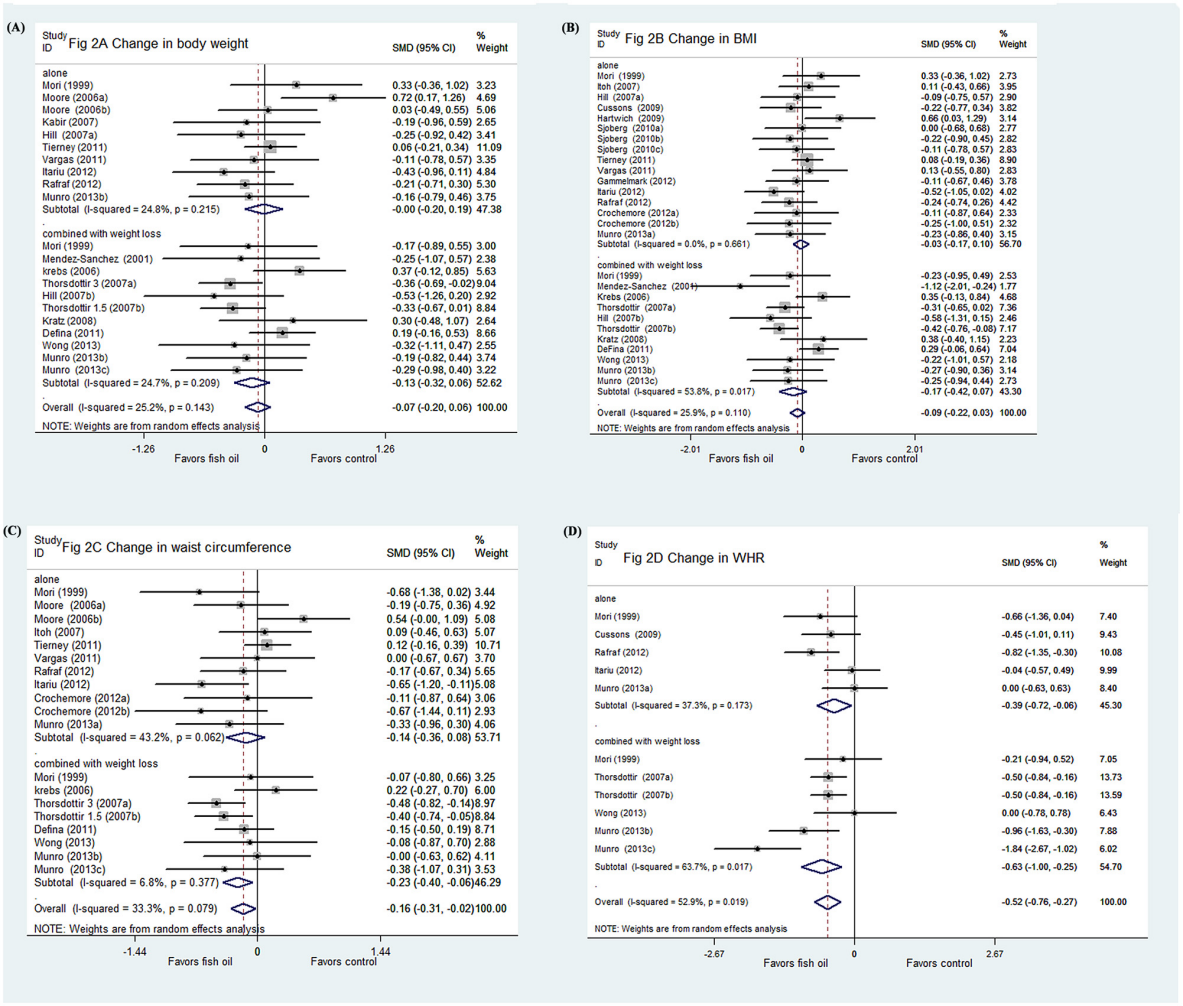
Forest plots from meta-analyses for the effects of fish oil on changes in body weight (A), BMI (B), waist circumference (C), and WHR (D).

In total, 21 comparisons with 1329 subjects investigated the effect of fish oil on body weight changes. No significant heterogeneity (I^2^ = 25.2%, *P* = 0.14) was found. The calculated results indicated that fish oil was not associated with a body weight reduction (SMD = -0.07, 95% CI -0.21 to 0.07, *P* = 0.31; Fig 2A) compared with controls.

Comparisons were divided into two independent analyses to assess whether fish oil in combination with life modification altered the analysis results. Of the studies, 10 comparisons [9, 18, 19, 22, 26–28, 31, 32] with 607 subjects investigated the effects of fish oil on changes in body weight alone. No significant heterogeneity (I^2^ = 24.8%, *P* = 0.22) was found. The calculated results indicated that fish oil was not associated with reduced body weight (SMD = -0.004, 95% CI -0.20 to 0.19, *P* = 0.97). Further subgroup analyses according to predefined study characteristics (including mean age, male proportion, total dose and ratio of EPA to DHA, follow-up duration, study design, and quality score) were also not statistically significant, which indicated that the relationship of fish oil supplementation with body weight changes seemed to not be influenced by these factors (Table 2).

**Table 2:** Subgroup estimation of the effects of fish oil on body weight changes.

**Change in Body weight**								
	**Alone**				**Combined with weight loss**			
	**Studies (subjects)**	**I** ^**2**^	**WMD [95%CI]**	***P***	**Studies (subjects)**	**I** ^**2**^	**WMD [95%CI]**	***P***
**Mean age (y)**								
≥50	6(415)	29%	0.13[-0.12,0.39]	0.31	3(92)	0%	-0.34[-0.77,0.09]	0.12
<50	4(192)	0%	-0.17[-0.45,0.12]	0.25	8(630)	41%	-0.09[-0.31,0.14]	0.46
**Male (%)**								
≥30	5(389)	37%	0.17[-0.12,0.45]	0.25	8(555)	20%	-0.19[-0.38,0.01]	0.07
<30	5(218)	0%	-0.17[-0.44,0.10]	0.21	3(167)	25%	-0.13[-0.32,0.06]	0.79
**BMI (kg/m** ^**2**^ **)**								
≥32	6(405)	0%	-0.01[-0.21, 0.19]	0.92	8(422)	8%	-0.02[-0.24,0.19]	0.84
<32	4(202)	57%	0.10[-0.33,0.54]	0.64	3(300)	18%	-0.28[-0.54,-0.02]	0.04
**Dose (g/d)**								
≥1.5	6(225)	0%	-0.10[-0.36,0.17]	0.46	11(722)	25%	-0.13[-0.32,0.06]	0.17
<1.5	4(382)	54%	0.13[-0.20,0.46]	0.44	—	—	—	—
**EPA/DHA**								
≥1	4(179)	0%	-0.17[-0.47,0.13]	0.26	2(153)	24%	0.07[-0.36,0.49]	0.25
<1	2(77)	0%	-0.21[-0.66,0.25]	0.38	4(204)	42%	-0.10[-0.51,0.31]	0.19
NR	4(351)	39%	0.23[-0.07,0.54]	0.13	5(365)	0%	-0.28[-0.49,-0.07]	0.64
**Duration (wk)**								
≥12	4(351)	39%	0.23[-0.07,0.54]	0.13	7(374)	17%	0.03[-0.22,0.27]	0.82
<12	6(256)	0%	-0.18[-0.43,0.07]	0.15	4(348)	0%	-0.32[-0.54,-0.11]	0.004
**Study design**								
DB	7(289)	0%	-0.12[-0.36,0.11]	0.31	10(697)	31%	-0.12[-0.32,0.08]	0.24
SB	3(318)	57%	0.23[-0.16,0.62]	0.24	1(25)	—	-0.32[-1.11,0.47]	0.43
**Score**								
≥3	9(574)	41%	-0.14[-0.37,0.08]	0.21	8(641)	41%	-0.13[-0.32,0.06]	0.21
<3	1(33)	0%	-0.07[-0.50,0.37]	0.77	3(81)	0%	-0.07[-0.50,0.37]	0.77
**Change in Body weight**								
	**Alone**				**Combined with weight loss**			
	**Studies (subjects)**	**I** ^**2**^	**WMD [95%CI]**	***P***	**Studies (subjects)**	**I** ^**2**^	**WMD [95%CI]**	***P***
**Mean age (y)**								
≥50	6(415)	29%	0.13[-0.12,0.39]	0.31	3(92)	0%	-0.34[-0.77,0.09]	0.12
<50	4(192)	0%	-0.17[-0.45,0.12]	0.25	8(630)	41%	-0.09[-0.31,0.14]	0.46
**Male (%)**								
≥30	5(389)	37%	0.17[-0.12,0.45]	0.25	8(555)	20%	-0.19[-0.38,0.01]	0.07
<30	5(218)	0%	-0.17[-0.44,0.10]	0.21	3(167)	25%	-0.13[-0.32,0.06]	0.79
**BMI (kg/m** ^**2**^ **)**								
≥32	6(405)	0%	-0.01[-0.21, 0.19]	0.92	8(422)	8%	-0.02[-0.24,0.19]	0.84
<32	4(202)	57%	0.10[-0.33,0.54]	0.64	3(300)	18%	-0.28[-0.54,-0.02]	0.04
**Dose (g/d)**								
≥1.5	6(225)	0%	-0.10[-0.36,0.17]	0.46	11(722)	25%	-0.13[-0.32,0.06]	0.17
<1.5	4(382)	54%	0.13[-0.20,0.46]	0.44	—	—	—	—
**EPA/DHA**								
≥1	4(179)	0%	-0.17[-0.47,0.13]	0.26	2(153)	24%	0.07[-0.36,0.49]	0.25
<1	2(77)	0%	-0.21[-0.66,0.25]	0.38	4(204)	42%	-0.10[-0.51,0.31]	0.19
NR	4(351)	39%	0.23[-0.07,0.54]	0.13	5(365)	0%	-0.28[-0.49,-0.07]	0.64
**Duration (wk)**								
≥12	4(351)	39%	0.23[-0.07,0.54]	0.13	7(374)	17%	0.03[-0.22,0.27]	0.82
<12	6(256)	0%	-0.18[-0.43,0.07]	0.15	4(348)	0%	-0.32[-0.54,-0.11]	0.004
**Study design**								
DB	7(289)	0%	-0.12[-0.36,0.11]	0.31	10(697)	31%	-0.12[-0.32,0.08]	0.24
SB	3(318)	57%	0.23[-0.16,0.62]	0.24	1(25)	—	-0.32[-1.11,0.47]	0.43
**Score**								
≥3	9(574)	41%	-0.14[-0.37,0.08]	0.21	8(641)	41%	-0.13[-0.32,0.06]	0.21
<3	1(33)	0%	-0.07[-0.50,0.37]	0.77	3(81)	0%	-0.07[-0.50,0.37]	0.77

Of the studies, 11 comparisons [16, 18, 23, 24, 26, 27, 30, 33, 52] with 722 subjects investigated the effects of fish oil on body weight changes combined with diet restriction and/or exercise. No significant heterogeneity (I^2^ = 24.7%, *P* = 0.21) was found among them. The calculated results indicated that fish oil had no additional weight loss effects (SMD = -0.13, 95% CI 0.32 to 0.06, *P* = 0.17). Subgroup analyses according to treatment duration demonstrated that studies with treatment less than 12 weeks favored significant weight loss effects of fish oil (*P* = 0.004). Sensitivity analysis excluded study with only 6 weeks of treatment [25], but they still suggested an insignificant relationship with fish oil and change in body weight (*P* = 0.22). Other predefined study characteristics were also not statistically significant (Table 2). Only one study included in this meta-analysis examined the combined effects of fish oil and regular exercise on body composition [18]. Sensitivity analysis excluding this trial did not seem to change the results (*P* = 0.28).

### Effects of fish oil on BMI changes

Of the studies, 27 comparisons with 1514 subjects investigated the effects of fish oil on BMI changes. No significant heterogeneity (I^2^ = 25.9%, *P* = 0.11) was found. The calculated results indicated that fish oil was not associated with a significant reduction in BMI (SMD = -0.09, 95% CI -0.22 to 0.03, *P* = 0.14; Fig 2B).

Among the studies, 16 comparisons [9, 15, 17–21, 26–28, 31–33] with 792 subjects investigated the effects of fish oil on changes in body weight alone. No significant heterogeneity (I^2^ = 0.0%, *P* = 0.66) was found. The calculated results indicated that fish oil had no effect in reducing BMI (SMD = -0.03, 95% CI -0.17 to 0.11, *P* = 0.64).

Of the studies, 11 comparisons [16, 18, 23–27, 29, 30, 52] with 722 subjects investigated the effect of fish oil on changes in BMI in combination with weight loss programs. The calculated results indicated that fish oil had no additional effect in reducing BMI (SMD = -0.17, 95% CI -0.42 to 0.07, *P* = 0.17) when combined with weight loss programs. In view of considerable heterogeneity (I^2^ = 53.8%, *P* = 0.02), subgroup analyses were performed to explore the potential difference between study characteristics and the effect of fish oil on changes in BMI. The results of subgroup analyses suggested that those with distinctive anti-obesity effects were studies with duration less than 12 week (*P* = 0.001), which could largely explain the considerable heterogeneity. After excluding the study with only 6 weeks of treatment [25], sensitivity analyses decreased heterogeneity (I^2^ = 46.3%, *P* = 0.06); however, the non-significant anti-obesity results remained (*P* = 0.32). Other factors including mean age, sex proportion, and total fish oil dose seemed irrelevant with the possible effects of fish oil on change in BMI combined with a weight loss program (Table 3). Furthermore, excluding the study that investigated the additional anti-obesity effect of fish oil combined with physical exercise [18] did not seem to change the results (*P* = 0.28).

**Table 3:** Subgroup estimation of the effects of fish oil on BMI changes.

**Change in BMI**								
	**Alone**				**Combined with weight loss**			
	**Studies (subjects)**	**I** ^**2**^	**WMD [95%CI]**	***P***	**Studies (subjects)**	**I** ^**2**^	**WMD [95%CI]**	***P***
**Mean age (y)**								
≥50	11(575)	0%	0.053[-0.11,0.22]	0.52	3(92)	0%	-0.35[-0.78,0.08]	0.12
<50	5(217)	0%	-0.18 [-0.43,0.08]	0.18	8(630)	65%	-0.13[-0.43,0.17]	0.39
**Male (%)**								
≥30	9(521)	0%	0.08[-0.10,0.25]	0.38	8(555)	45%	-0.17[-0.42,0.08]	0.19
<30	7(271)	0%	-0.18[-0.41,0.05]	0.13	3(167)	77%	-0.17[-0.42,0.07]	0.49
**BMI (kg/m** ^**2**^ **)**								
≥32	11(572)	0%	0.02[-0.14,0.18]	0.80	8(422)	54%	-0.16[-0.48,0.16]	0.34
<32	5(220)	0%	-0.11[-0.38,0.16]	0.42	3(300)	41%	-0.26[-0.58,0.06]	0.11
**Dose (g/d)**								
≥1.5	8(310)	0%	-0.05[-0.27,0.16]	0.64	11(722)	54%	-0.17[-0.42,0.07]	0.17
<1.5	8(482)	0%	0.01[-0.17,0.19]	0.89	—	—	—	—
**EPA/DHA**								
≥1	7(309)	0%	-0.15[-0.38,0.07]	0.18	2(153)	26%	0.16[-0.27,0.60]	0.25
<1	6(203)	0%	-0.15[-0.41,0.11]	0.26	4(204)	45%	-0.13[-0.55,0.29]	0.14
NR	3(280)	31%	0.26[-0.08,0.60]	0.13	5(365)	39%	-0.33[-0.64,-0.04]	0.16
**Duration (wk)**								
≥12	7(433)	0%	0.11[-0.08,0.30]	0.25	7(374)	29%	0.05[-0.22,0.32]	0.73
<12	9(359)	0%	-0.16[-0.36,0.04]	0.12	4(348)	0%	-0.40[-0.62,-0.18]	0.00
**Study design**								
DB	12(480)	0%	-0.05[-0.23,0.13]	0.69	10(697)	58%	-0.17[-0.43,0.09]	0.20
SB	4(312)	0%	0.04[-0.18,0.27]	0.83	1(25)	—	-0.22[-1.01,0.57]	0.59
**Score**								
≥3	10(587)	0%	-0.06[-0.22,0.11]	0.97	8(641)	65%	-0.22[-0.51,0.08]	0.15
<3	6(205)	14%	0.10[-0.19,0.38]	0.32	3(81)	0%	-0.03[-0.47,0.41]	0.89
**Change in BMI**								
	**Alone**				**Combined with weight loss**			
	**Studies (subjects)**	**I** ^**2**^	**WMD [95%CI]**	***P***	**Studies (subjects)**	**I** ^**2**^	**WMD [95%CI]**	***P***
**Mean age (y)**								
≥50	11(575)	0%	0.053[-0.11,0.22]	0.52	3(92)	0%	-0.35[-0.78,0.08]	0.12
<50	5(217)	0%	-0.18 [-0.43,0.08]	0.18	8(630)	65%	-0.13[-0.43,0.17]	0.39
**Male (%)**								
≥30	9(521)	0%	0.08[-0.10,0.25]	0.38	8(555)	45%	-0.17[-0.42,0.08]	0.19
<30	7(271)	0%	-0.18[-0.41,0.05]	0.13	3(167)	77%	-0.17[-0.42,0.07]	0.49
**BMI (kg/m** ^**2**^ **)**								
≥32	11(572)	0%	0.02[-0.14,0.18]	0.80	8(422)	54%	-0.16[-0.48,0.16]	0.34
<32	5(220)	0%	-0.11[-0.38,0.16]	0.42	3(300)	41%	-0.26[-0.58,0.06]	0.11
**Dose (g/d)**								
≥1.5	8(310)	0%	-0.05[-0.27,0.16]	0.64	11(722)	54%	-0.17[-0.42,0.07]	0.17
<1.5	8(482)	0%	0.01[-0.17,0.19]	0.89	—	—	—	—
**EPA/DHA**								
≥1	7(309)	0%	-0.15[-0.38,0.07]	0.18	2(153)	26%	0.16[-0.27,0.60]	0.25
<1	6(203)	0%	-0.15[-0.41,0.11]	0.26	4(204)	45%	-0.13[-0.55,0.29]	0.14
NR	3(280)	31%	0.26[-0.08,0.60]	0.13	5(365)	39%	-0.33[-0.64,-0.04]	0.16
**Duration (wk)**								
≥12	7(433)	0%	0.11[-0.08,0.30]	0.25	7(374)	29%	0.05[-0.22,0.32]	0.73
<12	9(359)	0%	-0.16[-0.36,0.04]	0.12	4(348)	0%	-0.40[-0.62,-0.18]	0.00
**Study design**								
DB	12(480)	0%	-0.05[-0.23,0.13]	0.69	10(697)	58%	-0.17[-0.43,0.09]	0.20
SB	4(312)	0%	0.04[-0.18,0.27]	0.83	1(25)	—	-0.22[-1.01,0.57]	0.59
**Score**								
≥3	10(587)	0%	-0.06[-0.22,0.11]	0.97	8(641)	65%	-0.22[-0.51,0.08]	0.15
<3	6(205)	14%	0.10[-0.19,0.38]	0.32	3(81)	0%	-0.03[-0.47,0.41]	0.89

### Effects of fish oil on waist circumference changes

In total, 19 comparisons with 1273 subjects investigated the effects of fish oil on changes in waist circumference. No significant heterogeneity (I^2^ = 33.3%, *P* = 0.08) among the patients was observed. The calculated results indicated that fish oil could significantly reduce waist circumference (SMD = -0.16, 95% CI -0.31 to -0.02, *P* = 0.03; Fig 2C).

Of the comparisons, 11 [9, 19–21, 26–28, 31, 32] with 649 subjects investigated the effects of fish oil on change in waist circumference alone. No significant heterogeneity (I^2^ = 43.2%, *P* = 0.06) was observed. The calculated results indicated that fish oil did not have a significant effect to reduce waist circumference alone (SMD = -0.14 95% CI -0.36 to 0.09, *P* = 0.22).

However, 8 comparisons [16, 24, 26, 27, 52] with 624 subjects investigated the effects of fish oil on change in body waist circumference combined with a weight loss program. The results determined that fish oil had a significant additional effect in reducing waist circumference (SMD = -0.23 95% CI -0.40 to -0.06, *P* = 0.008; I^2^ = 6.8%, *P* = 0.38) in combination with a weight loss program.

### Effects of fish oil on changes in waist hip ratio

Of the comparisons, 11 with 619 subjects investigated the effects of fish oil on changes in WHR. The calculated results suggested that fish oil could significantly reduce WHR (SMD = -0.52, 95% CI -0.76 to -0.27, *P* <0.0005; Fig 2D).

In total, 5 comparisons [15, 19, 26–28, 52] with 216 subjects investigated the effects of fish oil on change in WHR alone. The calculated results indicated that fish oil had a significant effect in reducing WHR alone (SMD = -0.39 95% CI -0.72 to -0.07, *P* = 0.02) without significant heterogeneity (I^2^ = 37.3%, *P* = 0.17).

Among the studies, 6 comparisons [26, 27, 30, 52] with 403 subjects investigated the effects of fish oil on changes in WHR combined with a weight loss program. The results also indicated that fish oil had a significant effect in reducing WHR when combined with a weight loss program (SMD = -0.63 95% CI -1.00 to -0.25, *P* = 0.001). However, there was significant heterogeneity (I^2^ = 63.7%, *P* = 0.02) among these studies. Subgroup analyses were not available for these outcomes because of the limited number of the studies included.

### Publication bias

**Fig 3:**
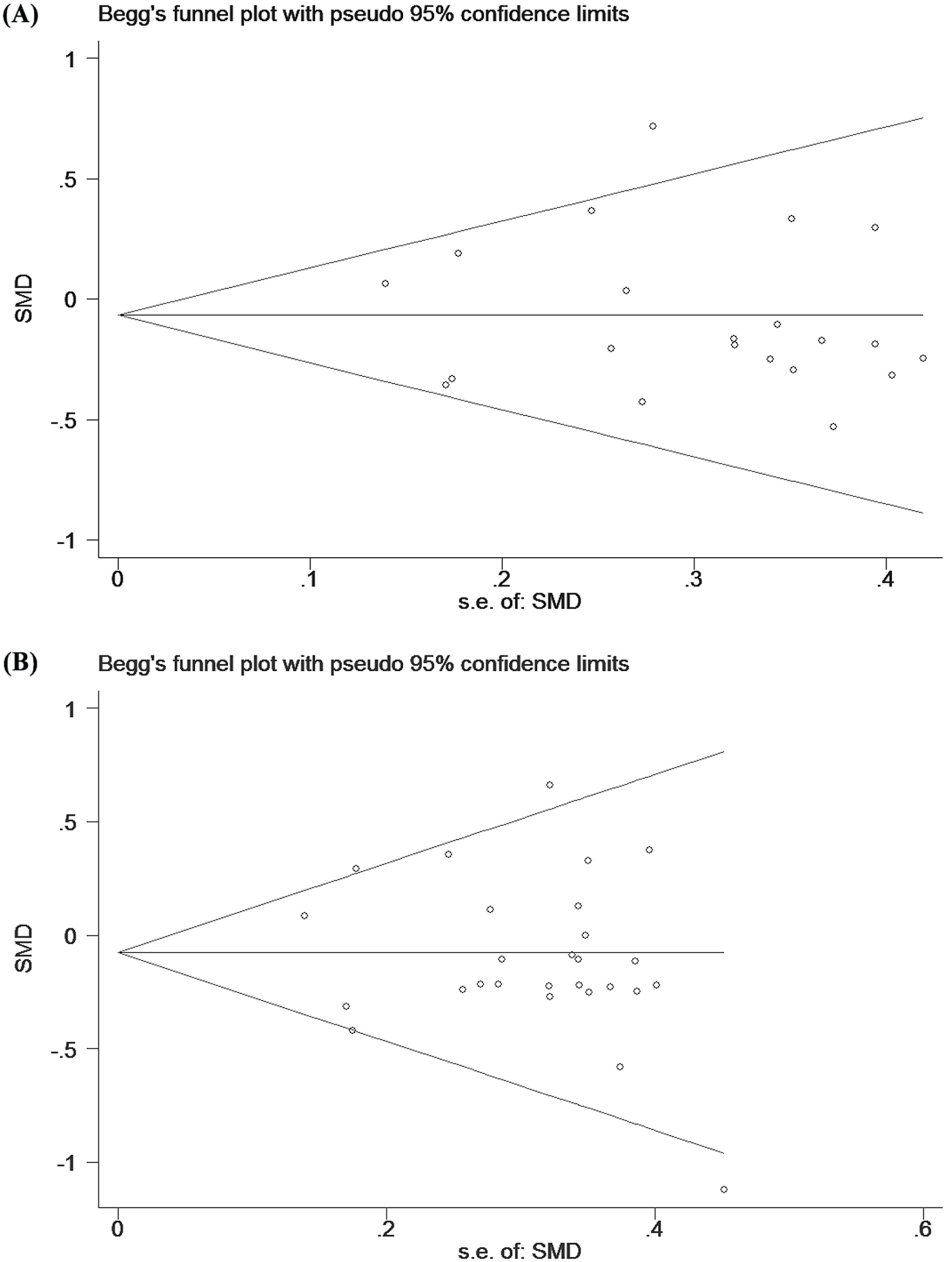
Funnel plots from meta-analyses for the effects of fish oil on changes in body weight (A) and BMI (B).

No significant publication biases were observed through funnel plots (Fig 3A and 3B) and Egger’s regression asymmetry tests for the effects of fish oil on changes in body weight (Egger’s test *P* = 0.79) or BMI (Egger’s test *P* = 0.18).

## Discussion

In the current meta-analysis, we investigated the association between changes in body composition and fish oil supplementation in 21 randomized, placebo controlled trials involving overweight or obese adults. Interestingly, in contrast to the results found in obese animal models, we did not detect a remarkable anti-obesity benefit of fish oil in overweight/obese subjects. However, fish oil might assist with improving waist hip ratio (WHR). We performed subgroup analyses to investigate the potential influences of study characteristics on pooled BMI and body weight outcomes. The results favoring significant weight reduction effects in the fish oil group combined with a weight loss program were those with treatment duration of less than 12 weeks. Furthermore, sensitivity analysis excluded the study that was only 6 weeks in duration, which demonstrated more stable negative results of fish oil supplementation compared with placebo. Gender differences have been considered as a potential factor to influence the effect of fish oil on reducing weight [27, 30, 40, 53]; however, in the subgroup analysis, we did not detect a significant difference with fish oil and weight reduction in different male proportions.

Munro et al [52] explained that perhaps n-3 PUFAs were more effective in preventing weight gain rather than assisting in weight loss. In humans, several studies have reported a significantly higher concentration of n-3 PUFAs in normal weight individuals compared with obese individuals [54, 55]. In addition, Couet C et al [56] observed that body fat mass was decreased and basal fat oxidation was significantly increased in 6 healthy volunteers with fish oil supplementation. However, Jakobsen et al [57] investigated 1998 lean adults and did not observe that the proportion of plasma n-3 PUFA was associated with a subsequent 1-year change in body weight. Large scale (both lean and obese subjects) and long term trials were needed to draw definite results.

Our results were supported by some previous observational studies. Garaulet et al [58, 59] investigated the n-3 PUFAs content of perivisceral and omental adipose tissue samples in 84 obese patients. Their study determined that n-3 PUFAs (in particular the DHA) were inversely related to abdominal obesity and adipocyte size. Matsumura et al [60] evaluated visceral fat by abdominal computed tomography in 91 subjects who had been fed with EPA 1800 mg/day and 74 control during a 6-month period. Their study observed that visceral fat area of male subjects in the EPA group trended to decrease (*P* = 0.06) during the 6 months of treatment. A number of pre-clinical and clinical studies [61–63] demonstrated an ameliorative effect of supplemental fish oil in reducing hepatic lipid content in non-alcoholic fatty liver disease (NAFLD). There were 2 review articles [64, 65] suggesting n3-PUFAs as potential treatments for liver inflammation associated with fat accumulation.

Because of the current limited information, we could not completely explain why fish oil decreased abdominal fat. However, increased n-3 PUFAs induced lipogenic gene down-regulation in adipose tissues. Second, it might have been related to changes in lipid synthesis and/or storage as a result of the profound reduction in postprandial lipemia associated with n-3 PUFAs [66]. Deck et al [67] reported that modest doses of fish oil supplementation caused a significant reduction in triglyceride levels by a mean of 2.21mmol/L and increased the high-density lipoprotein cholesterol by a mean of 0.13mmol/L. This was the first comprehensive meta-analysis to examine the effects of fish oil on changes of body composition in overweight and obese adults. We included only randomized controlled trials, which had a low probability of bias and other confounding factors of the original studies. Multiple electronic databases had been searched to minimize database bias. Selected trails had been carefully checked to minimize multiple publication bias. Individual search and extraction were conducted to minimize the bias in the provision of data and inclusion criteria.

Previous systematic reviews [68, 69] concluded that n-3 PUFAs might have potential benefits improving body composition in humans. However, their enrollment ranges (including lean and overweight/obese adults) were larger, and their study duration (3–12 weeks) less than ours. Furthermore, they did not use the meta-analysis method, which allow for greater statistical power than individual trials.

However, the results of this meta-analysis should be interpreted with caution because of several limitations. First, potential sources of heterogeneity included participant variations as well as intervention intensity and duration. The included trials had many differences including subject health status, concurrent therapy, fish oil supplement doses and compositions, follow-up durations, and treatment methods. These differences might have substantially contributed to the research heterogeneity. However, we expounded the influence of these factors through subgroup analyses. Second, treatment durations of the included study were relatively short (4 to 24 weeks), which made subgroup analysis according to study duration not compelling. Notably, plasma DHA levels increased significantly from 12 to 24 weeks [50]; however, in our meta-analysis, short- and long-term studies were assigned equal importance. We have limited information from RCTs on the relationship of body weight and fish oil in the longer term. Only 3 studies [9, 16, 24] included in this meta-analysis had treatment duration of 24 weeks. Tapsell et al [70] investigated the anti-obesity effects of fish diet for 12 month. However, the participants were in a “free living” state with self-reported food intake, and the dropout rate was as high as 47%. Some authors [27] suggested that it might be easier to manage dietary compliance when working with laboratory animals compared with humans. This might be an important reason underlying why the anti-obesity effects of fish oil supplementation were not as obvious in humans as animals.

In conclusion, from the results of our meta-analysis, we cannot obtain effective proof that fish oil intakes may decrease body weight in overweight/obese adults. However, it may help reduce the waist hip ratio especially when combined with life modification interventions. Because of the limited follow-up duration, the results should be treated with caution. Further large-scale research over a long time is needed to determine definitive conclusions.

## Supporting Information

S1 PRISMA Checklist (DOC)
